# “If I have teeth, I can smile.” Experiences with tooth loss and the use of a removable dental prosthesis among people who are partially and completely edentulous in Karnataka, India

**DOI:** 10.1038/s41405-021-00088-z

**Published:** 2021-09-13

**Authors:** Anisha Rodrigues, Shradha Dhanania, Rashmi Rodrigues

**Affiliations:** 1 Department of Prosthodontics, AJ Institute of Dental Sciences, Mangalore, India; 2 Department of Community Health, St. John’s Medical College, St. John’s National Academy of Health Sciences, Bangalore, India; 3 Global Public Health, Karolinska Institutet, Stockholm, Sweden; 4 DBT/Wellcome Trust India Alliance, Hyderabad, India

**Keywords:** Removable prosthodontics, Removable prosthodontics

## Abstract

**Objectives:**

Tooth loss is a phenomenon associated with structural and functional changes, with a negative influence on the oral and general health of an individual. Removable dental prosthesis is commonly fabricated as treatment for tooth loss. However, the perceptions and experiences of individuals during the transition from a dentate to a partially or completely edentulous state and their acceptance of the denture are largely ignored. The objective of this study was to explore the perceptions and experiences during the transition from a dentate state to being partially or completely edentulous in the Indian population.

**Methods:**

Fifteen individuals wearing partial or complete denture prosthesis were interviewed in depth using open-ended questions. These interviews were recorded using a voice-recording device. They were then transcribed, and a coding process was applied using the thematic framework approach to qualitative analysis.

**Findings:**

Five themes emerged, namely: (i) Transition from dentulous to partially or completely edentulous state; (ii) Varying experiences with the use of dentures; (iii) Convenience and duration of wearing dentures; (iv) Attitude of dentists towards patients’ complaints; (v) Knowledge and preference of available treatment modalities.

**Conclusions:**

The loss of natural teeth seemed to affect the psychological, functional, and social well-being of participants. Tooth loss was believed to be a natural aging process. Costs of treatment deterred the uptake of fixed denture treatment options. However, some participants preferred removable dentures to fixed prosthesis.

## Introduction

Edentulism (partial or total) is an irreversible oral health condition and a public health concern due to its economic burden and its impact on quality of life. It is influenced by risk factors such as age, sex, diet, oral hygiene, health behaviors, socioeconomic factors and level of education [1–3]. The most common causes of tooth loss are periodontal disease and dental caries. The rates of tooth loss are higher in Low- and Middle-Income Countries when compared to High-Income countries that have benefitted from scientific advancements in oral health.

The prevalence of edentulism in people above 50 years in India was estimated to be 16.3% [4], while the caries experience in the elderly was at 72%, with significant differences between the urban and rural population [5]. The prevalence of partial edentulism was 45.5% and increased toward the lower end of the socioeconomic spectrum [6].

Edentulism is a debilitating condition that manifests itself with structural and functional changes leading to a deleterious impact on oral and general health, decreased masticatory efficiency, poor facial appearance that adversely affects psychosocial well being and oral health related quality of life (OHRQOL) [7]. The impact on the OHRQOL however depends on the number, location and distribution of the teeth missing, that might affect chewing ability and esthetics [8]. Therefore, the perceptions of tooth loss may vary between individuals based on their personal, social and cultural beliefs, life experiences, adaptive capability, age, gender and educational level [9]. The general belief among the elderly that tooth loss is concomitant with the physiologic process of aging could influence their decision-making regarding preservation of teeth or their extraction. Moreover, though tooth loss is considered acceptable by some individuals, they could be unprepared for the physiological and structural changes that occur as a sequel, accompanied by negative emotions and a deep sense of loss [10].

High-Income Countries have echoed a change in social norms that have lead to a change in attitudes of patients towards healthcare. Improved access to oral health care and health information has enabled patients to engage in a more informed decision making in relation to their treatment needs. A study among 45–75 year old Irish patients on their preference towards treatment options for their partially edentulous state revealed high expectations and a high esthetic demand, preferring fixed replacements to removable dentures and conservation of natural teeth [11]. However, studies in Nigeria [12] and Northern India [13] have shown that patients were more concerned with the debilitating effects of tooth loss on mastication than on esthetics. There was higher acceptance of tooth loss and lower psychosocial impact compared to their Western counterparts.

The different treatment modalities for tooth loss depending on the number and location of missing teeth include implant-supported prostheses, fixed partial dentures (FPDs), removable partial dentures (RPDs) and complete dentures (CDs). While OHRQOL might not differ significantly between removable and complete denture wearers, it was reported that RPD wearers may face greater psychological and functional limitation than CD wearers who may have learnt to adapt to their handicap during their transition from a partially to a completely edentulous state [14].

Though there is sufficient literature on OHRQOL related to tooth loss and denture satisfaction, most of it is quantitative in nature with little flexibility to holistically capture patients’ perceptions. In this situation, qualitative research methods help explore, interpret and obtain a deeper understanding of patient experiences, attitudes and expectations. They might also inform why certain treatments or interventions work well with some patients, while with others they might not [15]. This understanding can aid an integrative treatment approach for successful treatment outcomes of the edentulous. This is especially so as dentists are required to adopt a patient-centric approach towards treatment planning that ensures the knowledge, needs, experiences and expectations of patients’ are acknowledged.

Further, given the transcultural differences in perceptions regarding tooth loss, translating findings from high-income contexts to India that has a diverse cultural background is not appropriate. It is therefore necessary, to understand the perceptions of the Indian population on tooth loss and the use of dentures for appropriate patient-centric treatment and improved OHRQOL. Patient perceptions regarding tooth loss and rehabilitation have implications not only for dentists but also for policy makers to bring about necessary changes in oral health policies regarding patient education, access and affordability of dental treatment [11, 16]. We therefore undertook this study to assess the perceptions surrounding tooth loss and life with a dental prosthesis.

## Methods

The study was conducted in the Department of Prosthodontics, A.J. Institute of Dental Sciences, Mangalore, Karnataka, South India. A.J. Institute of Dental Sciences is a tertiary care center and a dental teaching institute, located in an urban area. Dental treatment at the institute is provided at no-cost or at a nominal cost to all patients, depending on the type of treatment required. The Institute is also associated with the “Danta Bhagya Yojana” which is an oral health initiative by the Karnataka State government. The initiative provides free complete dentures to patients aged > / = 45 years and belonging to low-income groups. The institute therefore has a huge influx of patients, particularly from the low-and-middle income, not only from the city and surrounding areas, but also from the neighboring districts, despite the existence of large number of private dental clinics in the region.

This qualitative study involved in-depth interviews with 15 participants who either had partial or complete denture prosthesis. Purposive sampling ensured that male and female patients aged above forty years using a removable partial or complete denture with not more than twenty-four remaining natural teeth were included. The interviews were audio recorded using a voice-recording device and were conducted in a pleasant quiet room by SD and AR. They had no role in providing dental care to the participants. Eleven patients were interviewed individually till data saturation. We conducted four additional interviews to confirm that no new information emerged. A literature search along with expert consultation helped us develop questions that guided the interviews. The questions focused on specific domains such as experience of tooth loss, perceptions of denture use and alternate treatment modalities. Spontaneous and planned probes were used to obtain a deeper insight into the responses. The interviews were conducted in English or the local languages i.e., Kannada, Tulu or Hindi. Each interview lasted 30–40 min. The recorded interviews were transcribed verbatim and translated to English by an expert well versed in these languages as well as English. The transcribed data was then spot-checked by the authors to ensure consistency with the recordings.

### Data analysis

Transcribed data was analyzed using the thematic framework approach, wherein a subjective interpretation of the data was done through a systematic classification process of coding and identifying themes. The text was coded and the codes entered into a framework under which relevant data from the different transcripts was charted. Similar codes were brought under categories. Connections between the summaries were identified to form subthemes and overarching themes. This process was facilitated by discussions between the authors.

### Ethics statement

An approval for the study was obtained from the Institutional Ethics Committee. An informed consent was obtained from the participants prior to enrollment in the study. Verbal and written information regarding the aim, procedure and confidentiality of the study was given to all subjects.

### Findings

Overall, 15 partial and complete denture wearers participated in the study. They were aged 42–73 years, (mean age: 57.5years). Of these, seven were men and eight were women. Eight participants were partial denture wearers and seven were complete denture wearers. Twelve participants belonged to the lower socioeconomic group while three belonged to the middle socioeconomic group (Table 1).

**Table 1:** Participants’ sociodemographic characteristics.

Characteristic	Participants (*n* = 15)	
Age	57.5 (±9.7)	
Gender		
Male	7	46.7%
Female	8	53.3%
Education		
Primary	4	26.7%
High school	9	60%
Graduate	2	13.3%
Occupation		
Unemployed	4	26.7%
Unskilled	4	26.7%
Semiskilled	5	33.3%
Clerical/farm	2	13.3%
Socioeconomic status		
Lower/upper lower	12	80%
Lower middle	3	20%
Dental status		
Partially edentulous	8	53.3%
Completely edentulous	7	46.7%
Period Of denture wear		
<5 years	8	53.3%
5–10 years	2	13.3%
11–15 years	2	13.3%
16–20 years	2	13.3%
>20 years	1	6.6%

Socioeconomic status based on Kuppuswamy’s socio-economic status scale 2019.

Five themes emerged from the analysis, namely: (i) Transition from dentulous to partial or completely edentulous state; (ii) Varying experiences with the use of dentures; (iii) Convenience and duration of wearing dentures; (iv) Attitude of dentists towards patients’ complaints; (v) Knowledge and preference of available treatment modalities (Table 2).

**Table 2:** Themes and quotes.

Thematic framework components and quotes	Codes	Categories	Subthemes	Themes
Importance of oral health				
I feel it (tooth loss) is a normal process. What I feel is that it is due to age also, and maybe, I’m not cleaning properly.	Perceived notion that teeth will fall off with age	Age-related natural process	Perceptions of the tooth loss phenomenon	1
Problems without teeth				
I couldn’t smile properly during the healing period for around 20 days. I didn’t attend functions for sometime since it was my front teeth. When I lost my lower teeth, it didn’t affect much… I used to be careful while talking.	Self conscious about appearance and speech Did not socialize	Embarrassment in public	Psychosocial impact of edentulism	1
Denture experiences				
With the upper denture, I used to have pain. The skin used to peel off when I wore it. I used to find it difficult initially but later got used to it.	Painful ulceration Adapted over time	Denture problems	Initial adaptation to dentures	2
I can eat anything that you say. Even hard food. After going back home, I can video call and show you, or even make a video and upload it on YouTube.	Able to eat hard food Can prove it to anyone	Pleased with dentures	Benefits of wearing dentures	
I feel natural teeth are better. I feel the difference. Natural is always natural and it’s God gift…Food gets stuck in the denture but not in natural teeth.	Regret having to wear dentures	Unhappy with dentures	Disadvantages of dentures	
Denture usage				
I don’t feel it necessary to wear the denture at home. I mainly wear it to cover the hole when I smile.	Denture not worn at home	Denture use	Denture wearing habits	3
Dentists’ attitude				
I put denture in the mouth my words were not coming out properly. I told him that I do not like his work..so he replied that “this is how it will be.. whatever measurement you have given, it is according to that”. I did not wear the denture. I just used it for one or two days…I threw it... he made me a fool.	Not happy with the dentist’s attitude and work	Indifference of dentists towards patient complaints	Patient dentist relationship	4
Treatment options				
I feel the removing one is better. I feel it is easy to clean and I feel fixed dentures are very dangerous.	Better maintenance of removable dentures	Removable dentures better than fixed	Perceptions about removable dentures	5
I didn’t want fixed because I feel that I will have some problem because of it. I feel it will painful.	Afraid of fixed dentures Fixed dentures cause pain	Problems with fixed dentures	Deterrents for fixed denture treatment	

Themes: 1. Transition from dentulous to partial or completely edentulous state; 2. Varying experiences with the use of dentures; 3. Convenience and duration of wearing dentures; 4. Attitude of dentists towards patients complaints; 5. Knowledge and preference of available treatment modalities.

### Theme 1: Transition from dentulous to partial or completely edentulous state

Most participants had experienced tooth loss during their adolescence and early adulthood (20–35 years). While they initially lost one or two teeth, the number increased as they grew older. Tooth loss was attributed to lack of information on oral health care and preventive measures, and difficulty in availing dental services mainly due to poor accessibility and financial constraints. Teeth weren’t deemed important and participants resorted to home remedies for tooth pain, seeking professional care only when the situation worsened. They preferred to get their teeth extracted, as they believed that dentures were the best solution that would eliminate tooth related problems, were more economical and would require fewer future dental visits.

### Perceptions of the tooth loss phenomenon

The participants had varied perceptions and experiences with tooth loss. Some perceived tooth loss as an unavoidable age related natural phenomenon, and therefore wasn’t a serious issue. They also believed that as individual characteristics vary, so does tooth loss, depending on the individual’s systemic and risk factors.

“It (tooth loss) is natural. At around 50–80 years of age, only 50% of natural teeth remain. The rest, even if good, are 100% guaranteed to fall. It cannot be stopped by anyone.” (Male, Complete denture participant).

The participants acknowledged that lack of oral health knowledge, busy work schedules and time constraints, were reasons why they suffered dental decay, periodontal disease and eventual tooth loss. Some participants stated that their teeth were lost without the dentist’s intervention, mainly due to tooth mobility and “loose gums”. Oral hygiene was deemed important, and the lack of it affected social life.

“It is correct that teeth go away with age… but it is not natural to lose teeth in our age. We do not know how to clean properly. In front of the public we should look good. So, oral health is very important and we should maintain it. Bad oral hygiene will cause bad breath and also change the tooth color to yellow. We feel hesitant to talk to people.” (Female, Partially edentulous participant).

### Psychosocial impact of tooth loss

Though tooth loss was considered a normal occurrence, some participants expressed a sense of loss, as they felt that dentures were no match to natural teeth. The shape of the face seemed altered, speech was affected and difficulty with chewing, led them to avoid social gatherings. They eventually felt that natural teeth were “God’s gift.” Almost all participants expressed feelings of embarrassment and being self-conscious.

“I felt bad when they (friends) could talk and I couldn’t talk like them. It did not look good… Everybody, all friends made fun.” (Male, Partially edentulous participant).

However, missing teeth did not lead to embarrassment before their spouses, and such issues were considered a “wrong thinking process”.

Dentures helped restore the self-confidence of some participants, as they looked natural and improved their facial appearance. One participant explained:

“Now don’t I look good with the denture? Or else who would look at my face? When I used to go to a function, I used to take my plate separately. I used to hide behind the pillar and eat, so that people don’t see me eating.” (Male, Completely edentulous participant).

Teeth were considered important to function well at work:

“I go to some supply work, where everyone sees me. If I don’t have teeth, they might feel that I am not good looking. See, there is this actor Raj Kumar. He is 75 years old but he looks and acts like he is 55 because he has teeth.” (Male, Completely edentulous participant).

Tooth loss affected speech and phonetics due to involuntary movement of the tongue into the edentulous space. A religious woman felt that missing teeth affected her recitation of the Holy Book. The problem was resolved after wearing a denture.

Tooth loss affected diet and eating habits. Some could “never eat hard items”, could not eat quickly and therefore changed their food habits by having soft, over boiled or mashed food. Though they felt that tooth loss didn’t affect general health, some participants expressed their concern about indigestion due to inefficient chewing of food.

### Need for tooth replacement

The participants expressed the need for better esthetics, improved chewing and speech and therefore sought tooth replacement. One participant said he wanted to “reduce his age” and avoid being called “grandfather”. Some partially edentulous participants had to be coerced into getting dentures by their spouse, friends, children and even the dentist. Yet, they delayed treatment till they faced major issues or lost more teeth. Some participants chose to get their remaining teeth extracted and preferred complete dentures over partial dentures. One participant explained:

“Only one, two, three teeth were missing. I had no idea about the replacement… and treatment was not that advanced… there was no one to suggest me treatment options.” (Male, Partially edentulous participant).

### Theme 2: Varying experiences with the use of dentures

Irrespective of the type of denture (partial or complete), participants seemed to share similar experiences.

### Initial phase of adaptation to dentures

Participants reported negative experiences during the phase of initial adaptation to dentures, especially with mastication, denture retention, ulcerations, taste impairment, and increased salivation. However, over time, some issues were resolved.

“Initially, I didn’t feel any taste. Now, I am used to it. When I got the denture, it used to slip but now it’s fine.” (Male, Completely edentulous participant).

Many participants experienced difficulty in chewing and taking longer to eat with dentures. They complained of food lodgment beneath dentures and some partial denture wearers preferred to eat without dentures. Some felt that dentures had a “plastic feel” to them and were thick. One participant who could not adapt to his dentures and stopped wearing them said:

“I couldn’t bite. I couldn’t eat anything hard. Upper set used to fall down and lower set used to fly up. When I spoke loudly, the teeth would touch. So, now I think I’ll get a fixed one otherwise it will be the same again.” (Male, Completely edentulous participant).

### Benefits of wearing dentures

Dentures improved the appearance, speech and self-esteem of the participants, as they looked lifelike, were similar or even better than natural teeth and greatly helped with chewing.

“Denture gives strength to cheek… the pain also reduced…no problem to bite. It looks good…yellow color or gaps don’t look good… it (denture) also increases my confidence and makes me a look good.” (Female, Partially edentulous participant).

One of the participants explained how the denture enabled him to socialize with people:

“It is very good. I can go out. I can talk to people. I can sit in front of people. I can look at myself. I feel very happy. When I didn’t have teeth, people used to keep staring at my mouth.” (Male, Completely edentulous participant).

### Theme 3: Convenience and duration of wearing dentures

The partial denture participants chose to wear dentures to suit their comfort and necessity, mainly esthetics.

### Denture wearing habits

A few of the partial denture participants preferred to eat without dentures, as it took longer to eat with them, especially while in a hurry. Those who were comfortable without dentures did not wear them indoors. One of the respondents felt “weird” wearing dentures and wore them only when going out. On the contrary, some participants did not remove the dentures while traveling to other places, and wore them even while sleeping in the night. A participant who felt no one should know of his denture said:

“When I traveled to some other village, I did not take out the dentures at all. No one got to know. When I am at home, I take out the denture for 3–4 h a day as it wouldn’t be an embarrassment.” (Male, Completely edentulous participant).

### Spare dentures

Some participants preferred to have two sets of dentures, fearing accidental loss or breakage of the denture. One considered it a luxury to have two dentures, which he thought could be worn alternatively.

“It will be good if I have two sets, sometimes I can use this, sometimes that. Like if we have two cars, we can use both. Same way.” (Male, Completely edentulous participant).

### Theme 4: Attitude of dentists towards patients’ complaints

While most participants seemed very happy and satisfied with their dentist and the treatment they received, some were put off by the impatience and an indifferent attitude shown by the dentists due to which they were reluctant to disclose their problems. They chose to wear their ill fitting dentures fearing the dentist would ask them to get new dentures rather than fix their existing ones. One participant, unhappy with his denture seemed very upset with his dentist:

“The setting (denture) he gave was of plastic. I put it inside the mouth… words weren’t coming out properly. I told him that I did not like his work. He replied: ‘this is how it will be… whatever measurement you have given, it is according to that’. I did not wear it. I just used it for one or two days…I threw it… he made me a fool.” (Male, Completely edentulous participant).

### Theme 5: Knowledge and preference of available treatment modalities

There wasn’t much awareness about the treatment options among the participants, who mainly relied on the dentist to make decisions for them. Most participants were not aware of dental implants as treatment options.

### Perceptions about removable dentures

Though most participants were satisfied with their existing dentures, there were complaints of painful gums, food lodgment and unstable mandibular complete dentures. However, some preferred removable dentures to fixed dentures, as they were affordable, easy to clean and maintain. They seemed more practical as they could be removed and cleaned whenever required.

“I can take out these dentures and clean them. Even gums can be kept clean.” (Female, Partially edentulous patient).

### Deterrents for fixed denture treatment options

Increased cost of fixed dentures and implants were the main reasons to opt for removable dentures. Fear of pain involved was commonly cited by the participants.

“They said they will give me fixed teeth. But, I said I don’t want fixed because I was scared. If it starts hurting again, they will remove everything with pliers. So, I told them I don’t want fixed, I want removable.” (Male, Completely edentulous participant).

## Discussion

The findings of our study revealed the perceived need for tooth replacement for better esthetics and function. It also revealed the vulnerability of the participants to edentulism that resulted from negligence of oral health care due to a belief of inevitable tooth loss in the aging process. The participants in our study belonged to the lower socioeconomic group with a low educational level. Socioeconomic inequities are known to negatively influence oral health as people belonging to this group are more susceptible to dental caries, periodontal disease and subsequent tooth loss [9]. People within this group exhibit low oral health literacy, poor access to oral health and financial barriers to oral health care. In addition, the clinical management decisions by dentists might be different for this group due to treatment unaffordability. Dentists could possibly harbor negative perceptions of this group being ignorant and disregarding of their regular dental appointments and preventive practices.

Changes in appearance and difficulties with speech and chewing had a negative psychosocial impact on our participants. The emotions were complex, as they experienced embarrassment and low self-confidence, sadness and even resignation to mockery. It has been suggested that patients would be better prepared for the psychological effects of tooth loss if they had received advice from their dentists prior to teeth extraction. The participants expressed embarrassment of being seen without teeth in public. However, they did not feel embarrassed in front of their spouses as it was considered a natural aging process. Similar findings were noted in studies on elderly edentulous subjects in Iran and Hong Kong where strong cultural beliefs exist [17, 18]. In contrast, a study on edentulous patients in London stated that a majority of the patients avoided letting their partners see them without dentures [10].

Social and cultural factors have a very strong influence on the healthcare beliefs and practices in the Indian population. The general belief was that loss of teeth is a natural process, inevitable and unavoidable. This belief stems from a socio-cultural attribute linking the aging process with a decline in health [19]. Another common belief is that dentures are better than natural teeth as they require low maintenance, ensure permanent elimination of pain and other dental problems, and would minimize future dental visits, thereby reducing costs of care. Several studies have reported similar findings reflecting the participants’ feelings of resignation and acceptance of imminent tooth loss [9, 10, 18, 20].

Almost all our participants expressed difficulty in mastication due to tooth loss. The increased number of missing teeth led to a change in their dietary habits and food choices leading to consumption of soft food and sugary drinks, which in turn led to the problems of indigestion. Similarly, the study from Iran, reported that their participants believed improper chewing of food led to stomachache, distention and even nocturnal dyspnoea. Though they had initially adapted to tooth loss, they eventually faced a crisis [17]. Dental status appeared to influence food selection and nutritional intake leading to a poor nutritional status in older adults in UK [21].

Most participants in our study expressed great satisfaction with their dentures. Though they faced initial difficulties with their dentures, benefits outweighed problems. Only one of our participants was unhappy with his denture due to its poor retention and looked for fixed alternatives. A qualitative study in Brazil shares similar experiences of adaptation and acceptance of new dentures. The patients were greatly satisfied with treatment outcomes that provided functional and esthetic benefits [22]. Patient satisfaction with dentures may not correlate to the dentists’ evaluation of the prosthesis as patients are known to adapt to inadequate dentures, suggesting the influence of psychosocial and demographic variables with denture satisfaction. Less educated patients have exhibited greater denture satisfaction that is attributed to their lower levels of expectation. Also, higher economic status has been negatively correlated with denture satisfaction [23].

The patients’ preference for treatment options are known to largely depend on their knowledge regarding various treatment modalities and their financial concerns [24]. A higher literacy and educational level is associated with patient preference for fixed dentures [25, 26]. Though our Dental Institute offers treatment at no-cost to patients requiring RPD, CD and FPD, most of our participants preferred removable dentures due to their ease of maintenance, and the fear of pain with fixed dentures. Those who preferred implant supported prostheses, expressed costs as a major deterrent. A study on the knowledge and attitude towards prosthetic rehabilitation in Central India showed that a majority of patients had insufficient knowledge about the various treatment options. This knowledge was influenced by demographic factors like income, education, gender and age [27].

Though most patients expressed satisfaction with their dentist, not all patients felt the same. The attitude of the dentists towards denture patients could be considered as one of the gray areas that are seldom highlighted in edentulous patient care. In our study, the participants trusted their dentists in decision-making. Dentists should therefore build a good rapport and a trusting relationship with patients and understand their mental status before commencing treatment procedures [28]. It is also important to develop patience and soft skills for effective patient communication especially with denture wearers during their transition phase so that patients can better deal with the denture related problems. In addition, dentists should ensure continuity of care for their patients, paying heed to their sociocultural, psychological and economic situation. Relational continuity can be achieved through better communication and continued engagement. Being available for dental consultations whenever required ensures constinued care to the patients. This can reduce treatment costs, promote dental health, improve compliance to treatment as well as patient satisfaction thereby improving treatment outcomes [29]. Also, dentists can complement the public health care systems by creating oral health awareness, stressing on favorable oral health behaviors, the importance of oral rehabilitation and the treatment options for the same.

The participants in our study included both partial and complete denture wearers and explored how differently they perceived their edentulous and denture experiences. By large, they seemed to share similar experiences especially with their dentures. All participants experienced initial problems with adaptation to dentures. However, issues related to mastication and retention of dentures were more prevalent in complete denture patients. Eventually, most of them got accustomed to and were quite pleased with their dentures. RPD wearers chose to wear their dentures to suit their requirement and convenience.

The findings of our study are consistent with the findings of similar studies in other countries on participants belonging to the lower socioeconomic levels who expressed similar perceptions and experiences with tooth loss and rehabilitation. There is a need for measures to improve oral health awareness, reduce oral health inequalities, promote affordable dental health care and effective dentist–patient relationships particularly in the low-and middle-income countries.

### Strengths and limitations

The qualitative study design provided us greater flexibility in exploring and understanding the complex issues and situations related to tooth loss and the use of removable dentures. Unlike a quantitative assessment, the qualitative design facilitated an in-depth understanding of participant perceptions and experiences. Authors AR and SD, being prosthodontists as well as researchers, were able to effectively contextualize the responses and provide holistic interpretations of the findings. Both authors discussed the details of the interviews and the results at different stages of the analysis, resolving any conflict. Further assistance was obtained from author RR, who is experienced in mixed methods research. Thus, the credibility of the findings was ensured. However, the findings being driven by personal experiences, individual beliefs, socioeconomic and cultural backgrounds of the participants and the experiences of the researchers may not be reflective of a wider population. A description of our study context has therefore been provided in the methods section of this manuscript that would enable the reader judge the applicability of the findings to a different setting.

## Conclusion

This study provides an insight into the perceptions and experiences of people wearing removable dentures during the transition from a dentulous to an edentulous state, and finally to a dental prosthesis. Though tooth loss was considered a normal aging process, it affected their psychological, functional, and social well-being. Participants were greatly satisfied with their dentures despite the initial issues with its adaptation. It is essential for individuals to recognize the concept of maintenance of oral health at an early age and the need for appropriate dental rehabilitation when necessary to improve their oral health related quality of life.
